# Impact of an artificial intelligence-driven operational management system on operational efficiency in health care organization in Saudi Arabia: a mediating role of staff attitude

**DOI:** 10.3389/fpubh.2025.1558644

**Published:** 2025-04-30

**Authors:** Rakesh Kumar, Ajay Singh, Ahmed Subahi Ahmed Kassar, Mohammed Ismail Humaida, Sudhanshu Joshi, Manu Sharma

**Affiliations:** ^1^Department of Health Management, College of Public Health and Health Informatics, University of Ha’il, Ha’il, Saudi Arabia; ^2^Department of Management & Information Systems, College of Business Administration, University of Ha’il, Ha’il, Saudi Arabia; ^3^Department of Public Health, College of Public Health and Health Informatics, University of Ha’il, Ha’il, Saudi Arabia; ^4^School of Management, Doon University, Dehradun, India; ^5^Department of Management Studies, Graphic Era Deemed to be University, Dehradun, India

**Keywords:** artificial intelligence, health care, operational management system, staff attitude, operational efficiency, Saudi Arabia

## Abstract

**Introduction:**

In recent years, Artificial Intelligence (AI) is transforming healthcare systems globally and improved the operational efficiency in healthcare organizations. The authors examined how an artificial intelligence (AI)–driven operational management system (OMS) affected operational efficiency in health care units in the Kingdom of Saudi Arabia (KSA). They also investigated the mediating role of staff attitudes in the relationship between OMSs and operational efficiency. This research contributes to the field by applying the theory of planned behavior to examine health care professionals’ perceptions of AI-based OMSs and their impact on operational efficiency.

**Methods:**

To achieve study objectives, a quantitative research design, with cross-sectional survey methodology, was used to gather data from 287 health care professionals across various hospitals in the KSA. The authors used a partial least squares structural equation modeling (PLS-SEM) approach to hypothesis testing.

**Results:**

The findings indicated that an AI-based OMS significantly improved operational efficiency and positively affected staff attitudes. Furthermore, staff attitudes mediated the relationship between an AI-based OMS and operational efficiency.

**Discussion:**

The study finding highlights the dual benefits of AI-based OMSs in enhancing both operational performance and employee satisfaction. The results suggest that health care organizations in the KSA should invest in AI technologies to optimize operational efficiency and improve staff attitudes. The findings also emphasize the need to address employee perceptions to fully capitalize on the benefits of AI implementations. They also introduce staff attitudes as a mediating factor, offering new insights into the interaction between technology and employee engagement.

## 1 Introduction

Artificial intelligence (AI) integrated with health care management has revolutionized operational processes worldwide. It presents an unsurpassed opportunity for efficiency and enhancement in the delivery of services (1). Operational efficiency is the ability of an organization for optimizing processes with the least cost, time, and errors while maintaining quality. The Kingdom of Saudi Arabia (KSA) health care sector is driven by and in line with the Vision 2030 initiative (2). It has embraced AI-based operational management systems (OMSs) to enhance resource utilization, streamline patient care, and lower operational costs (3, 4). AI-based OMS refers to utilizing machine learning and predictive analytics to enhance and automate organization operations. This technology promotes better decision-making by refining workflows and boosting efficiency through real-time data analysis while minimizing the need for manual input. The use of AI is expected to contribute to decision-making processes, scheduling optimization, and patient flow management by improving operational efficiencies in the health care sector. It has several benefits for every industry around the globe. For example, AI has the potential to handle enormous data volumes and to automate routine tasks (5). Furthermore, AI can suggest predictive insights and skills that are important in a highly demanding area such as health (6). Thus, AI is regarded as a significant step to modernize the health sector in the KSA. It is in line with global trends relating to the adoption of this digital transformation in health care settings.

Employee attitudes have been identified in the available literature as central to any successful technology implementation. Employee attitudes encompass the perceptions, willingness, and apprehensions that workers have regarding the adoption of AI-driven OMS. It includes perceived utility, user friendliness, and reliance upon AI, as well as concerns about job loss due to automation. These factors significantly influence the successful adoption of the AI system. For example, attitudes toward new technologies taken up by staff can profoundly affect their effectiveness in health care units (7). In addition, positive perceptions and adaptability influence the degree of staff engagement with the AI systems (8). Moreover, staff could be resistant or show reluctance to use AI-based tools, which would automatically lower the potential of AI (9). The KSA has culturally distinctive and structured health care environments (10). An understanding of AI and its influence on operational efficiency, taking into account staff attitudes, is key to the successful integration of digital transformation in the health care operations in the KSA.

The adoption of AI-based OMSs in health care represents a great opportunity to enhance the efficiency of the entire health care sector in the KSA. The health care sector is considered to be among those undergoing modernization at one of the fastest rates in the KSA. Despite the fact that all these new technologies promise the optimization of resource utilization, cost reduction, and better patient care, but their effective implementation is not guaranteed (11). Furthermore, there is a human factor in this case: the attitude of health care staff toward an AI system which significantly affect the success or failure of such initiatives (12).

The health care sector of the KSA has some distinct features that may create challenges for the implementation of AI. It contains a very rigidly organized and hierarchically set environment (13, 14). Therefore, a resistance to or negative perception on the part of health care staff toward AI may undo the expected benefits and result in inefficiencies rather than improvements. Understanding this dual influence of AI systems and staff attitudes is important in maximizing their operational potential. Therefore, in this study we addressed the pressing need to evaluate not only the direct impact that AI itself makes on operational efficiency but also how staff attitudes mediate this relationship—a subject of utmost importance for the effective introduction of AI into health care.

We investigated the impact of an AI-based OMS on operational efficiency in health units within the KSA. We also addressed the mediating role of staff attitudes in the relationship between the AI-based OMS and operational efficiency in health care units in the KSA. We sought to answer the following research questions:

- How do AI-based OMSs in health care units in the KSA affect their operational efficiency?
- Do AI-based OMSs also influence staff attitudes toward work in health care units in the KSA?
- How do staff attitudes affect operational efficiency in health care units in the KSA?
- Do staff attitudes mediate the influence of AI-based OMSs on operational efficiency in health care units in the KSA?

This research provides timely and critical insights into the KSA’s rapidly changing health care landscape, driven by the ambitious Vision 2030 initiative. In a world where AI-based OMSs have become indispensable tools to enhance efficiency, understanding their real-world impact becomes paramount. The real significance of this study lies in its exploration of one of the most crucial, yet often overlooked, factors, including staff attitudes. AI systems promise great improvements in resource optimization and operational efficiency. However, their actual success is inextricably linked to how health care professionals perceive and engage with these technologies. We investigated the mediating role of staff attitudes as a means to progress from a purely technological perspective toward a holistic human–technology interface view in health care. The results will add to the increasing debate on AI in health. They also will add to practical knowledge for decision-makers in government and health leadership to notice or ensure meaningful, sustainable changes in operational efficiency. This doubled focus points to a very relevant study, both for the local and global health care system.

The key contributions of this study are substantial and exist on many fronts. First, the study adds to the growing body of literature on AI in the health care sector of the KSA. It provides initial empirical evidence of how AI-based systems can help enhance operational management. Much of the literature has focused on Western health care systems; this study addresses a critical gap in the literature by investigating the use of AI within a culturally distinctive non-Western setting. This research provides fresh insights into the ongoing global debate on AI’s transformative potential in health care.

Second, this study provides new insight into the mediating effect of staff attitudes in the relationship between AI-based OMSs and operational efficiency. Whereas most of the literature focuses on the core technology itself, this study brings the critical factor of human intervention into the limelight. It looks at how staff perceptions and attitudes can influence the effectiveness of the deployment of AI. Therefore, it can delve more deeply and holistically into how technology uptake will either work or fail because of human engagement.

Finally, this study contributes practically by providing real means through an approach by which health care leaders and policymakers can act. It emphasizes the development of positive attitudes among staff and the implementation of AI systems. It also helps ensure that technological adoption brings efficiency improvements that are sustainable at both the local and global health care system levels.

## 2 Literature review

### 2.1 Theoretical review

Several theories address the role of AI-based OMS on the efficiency of firms in diverse sectors in the literature. These theories include the technology acceptance model [TAM; (15)], diffusion of innovations (DOI) theory (16), and the theory of planned behavior [TPB; (17)]. The TAM is foundational to the understanding of technology adoption (18). It puts forward the hypothesis that perceived ease of use and perceived usefulness are critical determinants. The TAM explains that staff perceptions of AI systems’ usability and its benefits are central to its acceptance (19). Therefore, it is essential to realize operational efficiency in health care settings.

DOI theory gives insight into the spread of new technologies within organizations (20). Furthermore, DOI theory focuses on the overall propensity to adopt rather than the role of individual attitude in technology uptake (21). This makes it less comprehensive regarding the nuanced impact of staff attitudes on AI effectiveness. The Theory of Planned Behavior (TPB) elucidates behavioral factors by integrating attitudes, subjective norms, and perceived behavioral control (22). This framework effectively connects individual attitudes toward AI-based Operational Management Systems (OMS) with operational performance and implementation behavior, making it particularly relevant for examining AI implementation in the healthcare sector and beyond.

In comparison with models such as TAM and DOI, TPB best applies for the present study. TPB presents the most complete perspective of the psychological and social determinants of AI adoption and has effectively contributed to the study of organizational behavior. By embracing TPB, this research investigates staff perceptions as a mediator of the link between AI-based OMSs and operational effectiveness, providing useful implications for AI adoption in organizations.

### 2.2 Hypotheses

#### 2.2.1 OMSs and operational efficiency

Extensive research on the impact of AI-based OMSs in various sectors has proven to be very transformative. For instance, in manufacturing, AI systems have improved operational efficiency by optimizing production schedules and predictive maintenance that reduces downtime (23). Similarly, in the retail sector, AI inventory management has cleared a path to improving stock accuracy, reducing waste, and personalizing customer experiences to eventually drive efficiency and sales (24). With respect to logistics, AI algorithms have facilitated route planning and managing freight to result in greater cost efficiencies and speedier delivery times (25). AI also can automate tasks and analyze big data, resulting in massive efficiency gains across several industries (26).

AI also has massive potential in the health sector. For example, it can assist in optimizing patient scheduling and managing electronic health records (27). Furthermore, it can improve patients’ operational efficiency and performance using clinical decision-making assistants as subsections of health care organizations (28). Feretzakis et al. (29) stated that AI can predict admission rates and help achieve better resource disposition. Finally, it can also enhance the quality of diagnosis in imaging (30).

Despite these advances, there is a significant research gap on AI’s impact within the health care sector of the KSA. Most of the available studies have focused on Western contexts; hence, there is a significant level of ignorance about how AI systems perform in the KSA’s specific cultural and organizational context. This gap is of utmost importance because the KSA’s health care sector is modernizing rapidly under Vision 2030. Therefore, we proposed the first hypothesis (H1):

*H1*: An AI-based OMS significantly improves the operational efficiency of health care in the KSA.

#### 2.2.2 OMSs and staff attitudes

The literature provides valuable insights into the impact of AI-based OMSs on staff attitudes in all sectors. For example, the manufacturing sector indicates that AI systems usually bring about mixed reactions among staff (31). However, AI systems improve efficiency in operations with minimum levels of repetitive tasks (26). Furthermore, AI often raises concerns about job loss and changes in the scope of operations (32). Likewise, AI-driven tools increase job satisfaction by relieving employees of heavy manual retail work, such as inventory management and customer service (33). In contrast, employees also resist AI because it seems to surveil them and reduce their autonomy (34).

AI systems in logistics generally elicit positive attitudes among staff concerning navigation route planning and operation management (35). In these studies, employees have reported improved conditions and less stress because of AI. However, some findings indicate challenges in the acceptance of AI by staff because of the perceived complexity of the AI tools (36). AI has a somewhat different impact on the dimension of similarity in the health care sector. An AI system optimizes the scheduling of patients and the management of all electronic health records to support clinical decision making (27). Furthermore, it reduces many administrative burdens, leads to increased job satisfaction and allowing more time for patient care (37). However, concerns about the long learning curve and the reliability of AI in critical decisions create resistive attitudes among health professionals (36). There is a vast research gap regarding how AI-based OMSs influence the attitudes of staff within the KSA’s health sector. Most studies have focused on Western contexts, with little consideration for the unique cultural and organizational dynamics specific to the KSA. In light of the rapid modernization currently pursued by the health sector in the KSA in pursuit of Vision 2030, addressing this gap is now essential. An insight into how AI affects staff attitudes within this unique context will inform effective implementation, improved acceptance, and increased operational efficiency. Therefore, we formulated the second hypothesis (H2):

*H2*: Adopting an AI-based OMS has a positive impact on staff attitudes in health care in the KSA.

#### 2.2.3 Staff attitudes and operational efficiency

Staff attitudes are among the key influential variables in operational efficiency in many sectors (38). For example, manufacturing industries indicate that favorable staff attitudes presage better productivity and efficiency (39). Moreover, employees with favorable perceptions of their work environment also show proactive attitudes and behaviors related to improving operational performance (40). Conversely, negative attitudes result in low morale among employees, enhancing their turnover rates and perceivably generating operational disruptions and lower efficiency (41). In retail, staff attitudes toward technology and management practices is central. Research has identified that employees with positive attitudes are more productive, thus leading to better customer service (42). Furthermore, a positive attitude enhances teamwork, reduces errors, and smooth operations, hence influencing total productivity (43).

In health care, staff attitudes drive operational outcomes. For example, positive attitudes toward job roles and technology lead to better patient care and operational efficiency (44). That would be the case when health professionals have an overarching positive view of AI systems. This leads to efficient use of such systems, translating into efficiency and improved patient outcomes. There is an existing gap in research on how staff attitudes affect operational efficiency for health care entities in the KSA. Many of the relevant studies in this area have drawn on Western contexts and therefore lack the unique cultural and organizational dynamics at play in the KSA. This study will help formulate place-sensitive strategies that enhance operational efficiency in the pursuit of better health care delivery. Therefore, formulated the third hypothesis (H3):

*H3*: A positive staff attitude significantly improves the operational efficiency in health care in the KSA.

#### 2.2.4 Mediating role of staff attitudes

A positive attitude on the part of the staff mediates the impact of AI-based OMSs on operational efficiency across sectors. For example, it improves the effectiveness of technology adoption in manufacturing and retail sectors, improving the effectiveness of operational processes (45). Furthermore, a positive attitude on the part of staff fosters their acceptance of technology use and shapes operational outcomes (46). Studies have shown that health care professionals come forward to work with the technology of AI systems when their attitudes are more favorable. Therefore, the use of AI intensifies operational efficiency and improves patient care. In health care, staff attitudes mediate the impact of AI-based OMSs on efficiency through their influence on the adoption and use of technology. A positive attitude leads to better engagement with the AI tools (47). This is important in understanding the mediation process in the effective implementation of AI systems toward the desired realization of efficiency gains in health care settings. Therefore, we put forth the fourth hypothesis (H4):

*H4*: Staff attitudes significantly mediate the positive impact of AI-based OMS on the operational efficiency of health care in the KSA.

## 3 Materials and methods

### 3.1 Research design

We used a quantitative research design in this study, which comprised a cross-sectional survey. This involved taking data from large sample of health care professionals across various hospital departments in the KSA. A quantitative research design is used to obtain objective measurements and conduct statistical analyses of the relationships among variables (48). We sought to examine the impact of an AI-based OMS on operational efficiency in KSA health care units in a structured and systematic effort. We also ascertained the mediating role of staff attitudes. Therefore, a quantitative research design is appropriate for this study. The study also followed a positivist research philosophy. A positivist research study should be based on observable and measurable phenomena to ensure that its findings are evidence based and not a subjectively based interpretation (49). The phenomena in our study are measurable and observable and based on evidence. Therefore, the positivist research philosophy is appropriate for our study. Finally, we applied the deductive approach as the logical reasoning of our research. The deductive approach requires testing a theory on the basis of collected data. This allows for the opportunity to confirm or adjust the existing theories on the basis of the empirical evidence, adding to the robustness of the findings (50). In that regard, this study is structured and theory driven in analyzing the effect of AI systems on health care operational efficiency.

### 3.2 Population and sampling

The targeted population for this research involved health care professionals working in different hospital departments in Hail Health cluster in KSA at varying levels. The selection of this population gives a holistic view of the effect of an AI-based OMS on operational efficiency and the mediating role of staff attitudes in health care in the KSA. Including a wide array of professions helps one be certain that the findings reflect the real experiences and views of the individuals interacting with AI systems. The selection of a wide array of different professions within a specific population enhances the validity and relevance of the study (51). This target population was selected because of the leading role these professionals play in the daily activities of health care facilities. The doctors and nurses interact directly with both the patient care system and administrative systems. However, administrative staff manage processes and, if desired, can handle even the support side of integrating AI technologies. Therefore, pooling their inputs is thus crucial for understanding the broader implications of the AI systems for operational effectiveness.

We used a stratified random sampling strategy for data collection. Stratified random sampling considers the representation of different professional roles in health care departments (52). This approach increases the generalizability of the findings to various subgroups in the population, giving the researcher more specific details for the phenomena under study in diverse settings (53). Furthermore, stratified sampling minimizes sampling bias and enhances the reliability of findings (54). Ethical approval for the study was obtained from the Ethical Review Committee, University of Ha’il (Permission No. H-2024-351). Informed consent was obtained from all subjects involved in the study.

### 3.3 Research instrument and data collection

We relied on a structured questionnaire as the core research instrument to capture information about the impacts of an AI-based OMS, staff attitudes, and operational efficiencies for health care units in the KSA. The research questionnaire comprised four sections: demographic information, AI-based OMSs, staff attitudes toward such systems, and operational efficiency. The questionnaire was personally distributed to selected health care professionals across hospital departments in Hail Health cluster in the KSA. This method ensured direct and personal contact with the respondents, which improves the response rate and increases the validity of the data collected. The physical distribution of the questionnaire allows for immediate clarification of questions and thus ensures a greater degree of completeness in response (55).

However, for adequate statistical power for Structural Equation Modeling (SEM), Hair et al. (56) recommended at least 200–300 samples with the model complexity and number of model parameters being estimated varying accordingly. With these criteria, at least 250–300 responses were solicited for adequate model estimation. To cater for the likelihood of non-responses and for the study to be at least at the SEM sample requirement, a total number of 300 questionnaires were distributed. However, we got complete responses from 287 participants, indicating a response rate of 96%. This data collected sample met the SEM analysis minimum sample requirement for the estimation of robust and stable models.

### 3.4 Variable measurement

The questionnaire was based on established scales and adapted to the context of the study. Four items measuring an AI-based OMS were adopted from Yamin and Alharthi (57). Similarly, five items assessing staff attitudes were sourced from Pozzo et al. (58). Furthermore, seven items measuring operational efficiency scores were adopted from Zehir and Zehir (59). All of these constructs were measured using a 7-point Likert-type scale. The AI-based OMS, staff attitudes, and operational efficiency were the independent, mediating, and dependent variables, respectively. To facilitate participation, the questionnaire was made available in both English and Arabic. The provision of the questionnaire in these two languages ensures that respondents can comprehend and interpret the questions clearly. To ensure the content validity of the adapted items, the questionnaire was subjected to a pre-test conducted by domain experts. Seven specialists, comprising both AI professionals and healthcare professionals, evaluated the survey for clarity, content coverage, and overall comprehensiveness. Prior to its final use for data collection, a pilot study involving 32 healthcare professionals was carried out to evaluate the comprehensibility and reliability of the questionnaire, calculating Cronbach’s alpha to assess internal consistency.

### 3.5 Methods of estimation

We used the partial least squares structural equation modeling (PLS–SEM) as the estimation method in this study. In general, PLS–SEM is suitable for research that examines complex relationships between latent variables (56). In this study, we sought to discover how AI-based OMSs influence operational efficiency and the mediating role of staff attitudes. Therefore, PLS–SEM is appropriate for testing this study’s required set of hypotheses. PLS–SEM has considerable advantages for handling small sample sizes that fit the health care context set by the KSA. For example, direct and indirect effects can be estimated simultaneously using PLS–SEM, making it suitable for testing mediators (60). Furthermore, PLS–SEM is robust under violations of the normality in the distribution of data, which is usual in applied health care situations (61).

## 4 Results

Table 1 reports the summary of participants’ characteristics, such as their gender, marital status, age, education, job title, and nationality. These participants included 175 males and 112 females out of a sample of 287, representing 61, and 39%, respectively. Furthermore, the majority of these participants were married (186, or 64.8%). Furthermore, 127 participants were age 31–35, representing 44.3% of the sampled participants. Moreover, 62% of these participants had a bachelor’s degree. Their job titles indicated that the majority of these participants were doctors (135, or 47%). Similarly, the majority of these participants were Saudi (93, or 324%). The frequency and percentages of other categories of participants’ characteristics can be seen in Table 1.

**Table 1:** Summary of participant characteristics.

Category	*n*	%
Gender		
Male	175	61.0
Female	112	39.0
Marital status		
Single	101	35.2
Married	186	64.8
Age, years		
20–25	11	3.8
26–30	70	24.4
31–35	127	44.3
36–40	64	22.3
40+	15	5.2
Education		
Diploma	48	16.7
Bachelor’s	178	62.0
Master’s	29	10.1
Doctorate	32	11.1
Job category		
Doctors	135	47.0
Medical School Doctor	32	11.1
Administrators	52	18.1
Nurses	46	16.0
Paramedical staff	14	4.9
Allied services	9	3.1
Nationality		
Saudi	93	32.4
Egyptian	60	20.9
Sudanese	6	2.1
Indian	33	11.5
Pakistani	18	6.3
Filipino	72	25.1
Other	5	1.7

**Figure 1:**
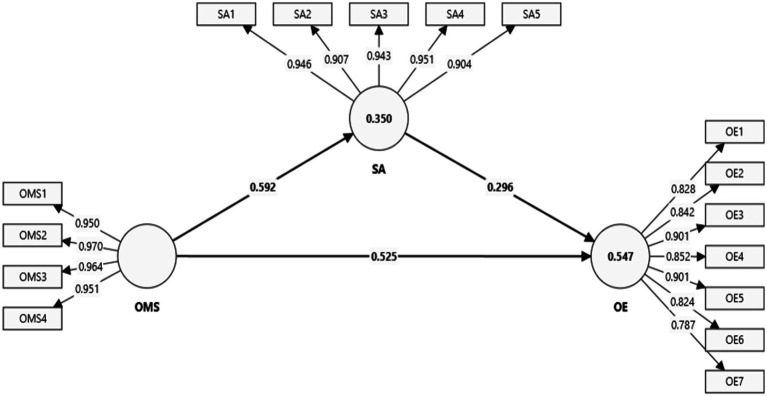
Measurement model. SA = staff attitude; OMS = operational management system; OE = operational efficiency.

Figure 1 depicts the study’s measurement model. The measurement model indicates how significantly accurately an indicator measures its construct (56). The measurement model also indicates the standardized factor loading values. According to Hair et al. (62) a factor loading of at least 0.70 or higher is required for an indicator to be considered as accurately measuring its construct. The reported values of indicators like OMS1–OMS4, SA1–SA5, and OE1–OE7 are greater than 0.70 (see Figure 1). Therefore, all the indicators accurately measured their relevant constructs: AI-based OMSs, staff attitudes, and operational efficiency.

Table 2 reports the constructs’ reliability in the form of convergent validity and reliability. *Convergent validity* refers to the fact that items or indicators of the measure of the same construct correlate, which shows they reflect the same underlying concept (63). *Reliability* in this respect refers to the consistency of items in the constructs, guaranteeing stability and repeatability of results across samples or time (64). Together, convergent validity and reliability confirm the appropriateness and dependability of a measurement model. According to Hair et al. (56), the measures used for establishing the convergent validity and reliability includes factor loadings, the variance inflation factor, Cronbach’s alpha, composite reliability, and average variance extracted (AVE). The thresholds are ≥0.70 for factor loadings, Cronbach’s alpha, and composite reliability; ≥0.50 or greater for AVE, and <5 for the variance inflation factor. The estimated values are under favorable limits for factor loadings, Cronbach’s alpha, composite reliability, AVE, and the variance inflation factor. Therefore, convergent validity, and the reliability of the constructs and indicators of this study, is confirmed.

**Table 2:** Convergent validity and reliability.

Constructs and their factors	FL	VIF	CA	CR (rho_a)	CR (rho_c)	AVE
Operational efficiency (OE)			0.935	0.937	0.947	0.720
OE1	0.828	2.175				
OE2	0.842	3.496				
OE3	0.901	3.658				
OE4	0.852	2.553				
OE5	0.901	2.328				
OE6	0.824	1.397				
OE7	0.787	2.576				
Operational management system (OMS)			0.971	0.971	0.978	0.919
OMS1	0.950	3.378				
OMS2	0.970	3.871				
OMS3	0.964	2.596				
OMS4	0.951	1.353				
Staff attitude (SA)			0.961	0.961	0.970	0.866
SA1	0.946	3.091				
SA2	0.907	2.891				
SA3	0.943	1.224				
SA4	0.951	3.216				
SA5	0.904	2.783				

FL = factor loadings; VIF = variance inflation factor; CA = Cronbach’s alpha; CR = composite reliability; AVE = average variance extracted.

*Discriminant validity* ensures that something is distinct from other constructs (65). Furthermore, it confirms that it measures something unique and is not overly correlated with other constructs (66). *Reliability* refers to a set of items that applies consistency and stability in measuring the same construct to ensure replicability of the results across time or different samples (64). Together, they ensure that a measurement model precisely captures unique constructs reliably. Tables 3–5 report the discriminant validity in the form of heterotrait–monotrait (HTMT) ratios, the Fornell–Larcker criterion, and cross-loadings.

**Table 3:** Heterotrait–monotrait (HTMT) ratios.

	OE	OMS	SA
OE			
OMS	0.731		
SA	0.636	0.612	

OE = operational efficiency; OMS = operational management system; SA = staff attitude.

**Table 4:** Fornell–Larcker criterion.

	OE	OMS	SA
OE	0.849		
OMS	0.700	0.959	
SA	0.606	0.592	0.930

OE = operational efficiency; OMS = operational management system; SA = staff attitude.

**Table 5:** Cross-loadings.

Indicator	OE	OMS	SA
OE1	0.828	0.533	0.500
OE2	0.842	0.543	0.448
OE3	0.901	0.573	0.480
OE4	0.852	0.601	0.510
OE5	0.901	0.666	0.581
OE6	0.824	0.618	0.558
OE7	0.787	0.602	0.504
OMS1	0.660	0.950	0.594
OMS2	0.674	0.970	0.585
OMS3	0.665	0.964	0.551
OMS4	0.685	0.951	0.538
SA1	0.580	0.554	0.946
SA2	0.559	0.522	0.907
SA3	0.555	0.561	0.943
SA4	0.566	0.563	0.951
SA5	0.562	0.552	0.904

OE = operational efficiency; OMS = operational management system; SA = staff attitude.

Table 3 reports HTMT ratios as the first criteria to establish the discriminant validity and reliability of the construct. According to Hair et al. (56), the threshold includes an HTMT ratio <0.90 between two different constructs. The estimated HTMT ratio is as per the defined criteria. Therefore, validity and reliability, as assessed using the HTMT ratio, are confirmed for all the constructs of this study: AI-based OMS, staff attitude, and operational efficiency.

Table 4 reports the Fornell–Larcker criterion to assess the discriminant validity and reliability of constructs. According to Hair et al. (56), the square root values of AVE for any construct should be greater than its square root values with other constructs. The estimated values of the AVE square root are higher in case of its own correlated values, whereas correlated values of the same are less in case of other constructs. Therefore, validity and reliability as assessed using the Fornell–Larcker criterion is confirmed for all the constructs of this study.

Table 5 reports the cross-loading values for all the indicators and under their own constructs, as well the other constructs of this study. According to Hair et al. (56), the cross-loading values of indicators for their own construct should be significantly higher compared with their cross-loading values in other constructs. The reported cross-loading values are significantly higher for indicators in case of their own construct. Moreover, the cross-loading values are significantly lower for indicators in case of other constructs. Therefore, discriminant validity and reliability as assessed using cross-loading criteria is confirmed for all the constructs of this study.

Table 6 reports the model fit indices using PLS–SEM estimations. According to Hair et al. (56), the standardized root-mean-square residual value should be <0.08, and the normed fit index should be close to or greater than 0.90 to confirm the model as statistically fit. The reported standardized root-mean-square residual values, and the normed fit index in the case of the saturated model, as well as in the case of the estimated model, meet the criteria. Therefore, the study’s model is statistically fit.

**Table 6:** Model fit.

Index	Saturated model	Estimated model
SRMR	0.055	0.055
d_ULS	0.408	0.408
d_G	0.688	0.688
*χ* ^2^	1,058.177	1,058.177
NFI	0.926	0.926

SRMR = standardized root-mean-square residual; d_ULS = squared Euclidean distance; d_G = Geodesic distance; NFI = normed fit index.

**Figure 2:**
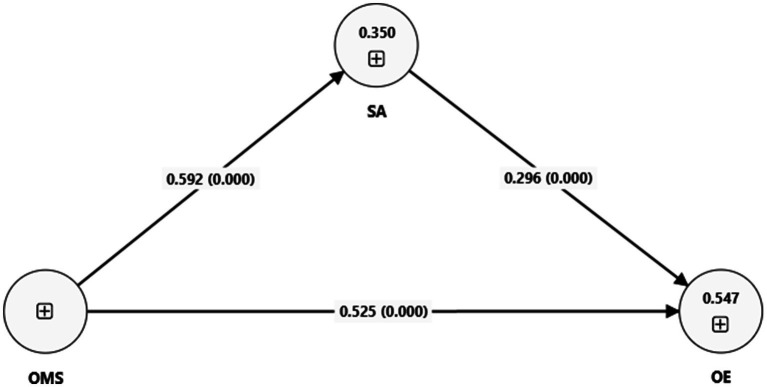
Structural equation model. SA = staff attitude; OMS = organization management system; OE = operational efficiency.

Figure 2 reports the structural equation model in graphical mode that was generated using Smart PLS software (Version 4). It indicates a positive and significant impact of an AI-based OMS on operational efficiency. Similarly, it also shows a positive and statistically significant impact of an AI-based OMS on staff attitude. Moreover, the figure also indicates a positive and statistically significant impact of staff attitude on operational efficiency.

Table 7 reports the estimated of structural equation model (SEM) in the form of coefficients, standard deviations, *t* values, and *p* values.

**Table 7:** Structural equation model (SEM) estimates.

Relationships	Coefficient	*SD*	*t*	*p*	Decision
OMS → OE	0.525	0.054	9.743	0.000	H1: Supported
OMS → SA	0.592	0.032	18.329	0.000	H2: Supported
SA → OE	0.296	0.060	4.942	0.000	H3: Supported
OMS → SA → OE	0.175	0.037	4.746	0.000	H4: Supported

H1 = first hypothesis; H2 = second hypothesis; H3 = third hypothesis; H4 = fourth hypothesis; OMS = organization management system; OE = operational efficiency; SA = staff attitude.

An SEM analysis indicated a positive impact of an AI-based OMS on operational efficiency. The coefficient value of this impact is β_1 = 0.525 with a *p* value of 0.000. The finding suggests the important impact of AI systems in enhancing operational efficiency in the KSA’s health care sector. This finding supports our hypothesis that AI-based OMSs enhance operational efficiency in the KSA’s health care sector.

An SEM analysis also indicated a positive impact of an AI-based OMS on staff attitude. The coefficient value of this impact is β_2 = 0.592, with a *p* value of 0.000. This finding supports our hypothesis that an AI-based OMS improves the staff attitude in the KSA’s health care sector.

The table also reports a positive impact of staff attitude on operational efficiency. The coefficient value of this impact is β_3 = 0.296 with a *p* value of 0.000, indicating that favorable staff attitudes contributed to good operational efficiency.

Finally, Table 7 reports the mediating role of staff attitude for the impact of an AI-based OMS on operational efficiency. The coefficient value of this impact is β_4 = 0.175 with a *p* value of 0.000. Because this coefficient is positive, it indicates partial mediation by staff attitude in the relationship of an AI-based OMS with that of operational efficiency. This result supports the hypothesis that staff attitudes play an important mediating role in the effect of AI systems on operational efficiency. The suggests that although AI systems enhance operational efficiency directly, part of this direct effect is mediated through improvements in the attitude of the staff.

## 5 Discussion

In this study, we assessed health care professionals’ opinions of the impact of AI-driven OMSs on health care organizational efficiency. The study yielded the following four results: (a) an AI-based OMS strongly increases the operational efficiency in health care in the KSA, (b) an AI-based OMS significantly improves staff attitudes, (c) positive staff attitudes in health care in the KSA also significantly enhances operational efficiency, and (d) health care staff attitudes partially and significantly mediate the positive impact of an AI-based OMS on operational efficiency.

The result that an AI-based OMS strongly enhances operational efficiency supports similar findings in the literature indicating that AI technologies enhance operational procedures by increasing data accuracy, smoothing workflows, and facilitating more informed decisions. This finding is consistent with those of several previous studies (27–30). The evidence confirms the practical benefit of adopting AI systems in health care and reinforces their value in enhancing operational efficiency in the KSA’s health care sector. In addition, the findings of this study verify that AI systems have improved operational efficiency by optimizing production schedules and predictive maintenance that reduces downtime (23). Similarly, AI inventory management has cleared a path to improving stock accuracy, reducing waste, and personalizing customer experiences to drive efficiency (24). Likewise, AI algorithms have facilitated route planning and supply chain management, resulting in greater cost efficiencies and speedier delivery times (25). Moreover, AI systems contribute to operational efficiency in the KSA’s health care sector by automating routine tasks and reducing manual errors (26). All of the studies we have cited were conducted in either a Western setting or in a different sector, which created research gap in the KSA’s health care setting that this study aims to fill. Hence, all of the previous evidence verifies the positive impact of AI-based OMSs on operational efficiency. Therefore, AI-based OMSs play a positive role in enhancing the operational efficiency in health care in the KSA.

An AI-based OMS significantly improved staff attitudes in the KSA’s health care sector. This finding is consistent with those of several previous studies (27–35). Most of these studies have focused on Western contexts, with little consideration given to the unique cultural and organizational dynamics specific to the KSA. In light of the rapid modernization currently pursued by the health sector in the KSA in pursuit of Vision 2030, it was essential to address this gap. The findings of this study provide insight into how AI affects staff attitudes within the unique context that will inform effective implementation, improved acceptance, and increased operational efficiency.

The evidence shows the extent of AI systems in not only operational efficiencies but also in creating a better and friendlier work environment in health care. Therefore, it highlights dual benefits in the KSA context. This result is also consistent with research showing how AI can have a positive effect on employees’ attitudes by alleviating their administrative burden (26). AI equips health care staff with superior means of performing their jobs and improving communication at work (32). Hence, in line with previous studies, the findings of this study suggest that an improved staff attitude toward AI systems makes processes more efficient and less prone to errors. Therefore, AI may enhance job satisfaction and reduce stress for health care workers, which ultimately enhances operational efficiency in health care.

Positive staff attitudes in the health care field in the KSA also significantly enhance operational efficiency. This result supports the hypothesis that staff attitudes play a very important role in enhancing operational efficiency in health care units in the KSA. This result is also consistent with the results of several previous studies. For example, staff attitudes are among the key influential variables in operational efficiency in many sectors (38). In addition, research focused on manufacturing industries indicates that favorable staff attitudes presage better productivity and efficiency (39). Moreover, the people with favorable perceptions of their work environment also show proactive attitudes and behaviors for improving operational performance (40). Likewise, negative attitudes result in low morale among employees, increasing turnover rates and generating perceptible operational disruptions and lower efficiency (41). In retail sectors, the attitudes of staff toward technology and management practices is central. Research has identified that employees with positive attitudes are more productive, thus leading to better customer service (42). Furthermore, a positive attitude enhances teamwork, reduces errors, and smooth operations, hence influencing total productivity (43).

Similarly, positive attitudes among staff members can lead to higher levels of job performance and productivity and the effective utilization of resources (44). Improved attitudes lead to better collaboration, reduced errors, and a more efficient working environment (67). Hence, in line with previous studies, the findings of this study suggest that improved staff attitudes toward AI systems make processes more efficient and less prone to errors. In the setting of the KSA’s health care sector, where personnel engagement has been highlighted as the bedrock of operational success, this evidence underlines the development of positive staff attitudes as a prime driver of efficiency. This shows that there is an interrelationship between human factors and a technologically advanced environment that facilitates excellence in operations.

Health care staff attitudes partially and significantly mediate the positive impact of AI-based OMSs on operational efficiency. The literature indicates that a positive attitude on the part of the staff mediates the impact of AI-based OMSs on operational efficiency across sectors. For example, employees’ attitudes and perceptions can make a difference in terms of the impact of technological change on general performance (68). Moreover, they improve the effectiveness of technology adoption in manufacturing and retail sectors, improving the effectiveness of operational processes (45). Furthermore, a positive attitude on the part of staff fosters employees’ acceptance of technology use and shapes operational outcomes (46). These studies prove that health care professionals are more willing to work with AI systems when their attitudes are more favorable. Therefore, the use of AI intensifies operational efficiency and improves patient care. In the health care field, staff attitudes mediate the impact of AI-based OMS on efficiency through their influence on the adoption and use of technology. Finally, a positive attitude leads to better engagement with AI tools (47). These findings, in the context of the KSA’s health care sector, signal that it is important not only to implement AI systems but also to maintain good, positive staff attitudes if the benefits of technology for operational performance are to be fully realized.

These results reinforce the robustness and universality of TPB for explaining AI implementation within the operations in healthcare organizations. The outcome conforms with the study goals by exhibiting that operational efficiency is highly determined by attitudes toward AI-based OMSs. The study showed that perceived control of behavior contributed toward the decision of the personnel using the AI systems, upholding the TPB’s universality. Despite the anticipation that subjective norms were likely to be the most influential factor, the impact of the latter proved moderate, implying that employee personal assessment of the benefit of AI may override external forces of social pressure.

### 5.1 Study implications

These findings also have crucial theoretical implications that are based on the TPB, which postulates that attitudes, subjective norms, and perceived behavioral control influence intentions and behaviors. This study extends the TPB by showing how AI-based OMSs affect staff attitudes and, hence, operational efficiency, in health care settings. The positive effect of AI-based OMSs on staff attitudes justifies the TPB’s assumption about the impact of attitudes toward a particular behavior on behavioral outcomes. In this regard, the mediation analysis in this study provides evidence that improved staff attitudes mediate the relationship between AI systems and operational efficiency. This is an indication that a positive attitude serves a crucial role in upgrading the effectiveness of technology adoption. In general, this study enhances the TPB by embedding technology adoption into the framework, and it pinpoints the role of attitude in leveraging technological capabilities for better organizational outcomes.

The findings of this study have several practical implications for the health care sector in the KSA. First, the enormous improvement in operational efficiency by means of an AI-based OMS draws the attention of health care organizations toward the need to implement such technology. AI will thus help speed up processes, enhance data accuracy, and allow better resource management, thereby boosting efficient health care delivery. This again suggests that addressing employee perceptions and engagement is also a relevant factor in the adoption of technology, because AI systems have been observed to positively influence staff attitudes. Health care leaders should now institute thorough training and support to make staff comfortable and willing to work with AI tools. The study indicates that positive staff attitudes can enhance the benefits of AI systems. Therefore, it suggests that developing a supportive work environment is crucial. Investing in strategies for improving morale and increasing job satisfaction will yield better outcomes from the operations and lead to improved efficiency and higher quality health care services that use AI.

### 5.2 Limitations and suggestions for future research

This study has several limitations. First, the cross-sectional nature of these data means they captured information at only one point in time, so changes over time, or causality, cannot be deciphered. Longitudinal studies could provide more insight into the impact of AI systems on operational efficiency and staff attitudes over the longer term. In addition, this research targeted health care units only in the KSA; this limits generalization of the results to other regions or sectors. Future research can examine similar relationships in different cultural and organizational contexts to make the findings more generalizable. In addition, the study addressed the most critical variables. Other elements could influence the impact of AI systems. For example, organizational culture and leadership style could be considered in future research. These are variables that, in the future, could be regarded as getting a holistic view of how AI will affect health care operations. Last, the sample size could be increased and the study extended to diverse health care settings to further instantiate the results with a more nuanced view of the effects of AI.

## 6 Conclusion

The results of this study offer detailed insights into the impact of AI-driven Operational Management Systems (OMS) on both operational efficiency and staff attitudes within healthcare facilities in Saudi Arabia. The findings indicate that AI-based systems significantly enhance operational effectiveness. Consequently, AI plays a crucial role in optimizing healthcare operations by streamlining processes and improving data accuracy. Additionally, the research demonstrates that the implementation of AI-driven systems affects personnel attitudes, significantly influenced by individual perception of these systems.

The study results further confirm that the implementation of advanced technologies enhances operational efficiency and improves worker satisfaction and engagement. The mediation analysis revealed that workers’ attitudes significantly mediate the relationship between AI-based OMS and operational efficiency, thereby validating the Theory of Planned Behavior (TPB) model for AI adoption in the healthcare sector. The study contributed to the theoretical extension of TPB by adding AI-based technologies within the model and emphasizing attitude development for the use of the technology for operational improvement.

## Data Availability

The raw data supporting the conclusions of this article will be made available by the authors, without undue reservation.
